# 6-(4-Bromo­phen­yl)-2-(4-fluoro­benz­yl)imidazo[2,1-*b*][1,3,4]thia­diazole

**DOI:** 10.1107/S1600536811007343

**Published:** 2011-03-05

**Authors:** Afshan Banu, Noor Shahina Begum, Ravi S. Lamani, I. M. Khazi

**Affiliations:** a Department of Chemistry, Bangalore University, Bangalore 560 001, India; b Department of Chemistry, Karnatak University, Dharwad 580 003, India

## Abstract

In the title compound, C_17_H_11_BrFN_3_S, the imidazothia­diazole and bromo­phenyl rings are individually almost planar, with maximum deviations of 0.0215 (4) and 0.0044 (4) Å, respectively, and are inclined at an angle of 27.34 (3)° with respect to each other. The dihedral angle between the mean planes of the fluoro­benzyl and imidazothia­diazole rings is 79.54 (3)°. The crystal structure is stabilized by inter­molecular C—H⋯N inter­actions resulting in chains of mol­ecules along the *b* axis.

## Related literature

For general background to imidazothia­diazole derivatives, see: Palagiano *et al.* (1995 ▶). Accumulation of fluorine on carbon leads to increased oxidative and thermal stability, see: Strunecka *et al.* (2004 ▶); Park *et al.* (2001 ▶). For related structures, see: Yang *et al.* (2006 ▶).
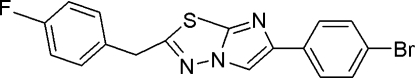


## Experimental

### 

#### Crystal data

C_17_H_11_BrFN_3_S
*M* *_r_* = 388.26
Monoclinic, 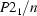

*a* = 10.505 (4) Å
*b* = 5.617 (2) Å
*c* = 25.877 (11) Å
β = 91.566 (7)°
*V* = 1526.2 (11) Å^3^
*Z* = 4
Mo *K*α radiation
μ = 2.84 mm^−1^
*T* = 296 K
0.18 × 0.16 × 0.16 mm

#### Data collection

Bruker SMART APEX CCD detector diffractometer
Absorption correction: multi-scan (*SADABS*; Bruker, 1998 ▶) *T*_min_ = 0.629, *T*_max_ = 0.659
8589 measured reflections
3308 independent reflections
2419 reflections with *I* > 2σ(*I*)
*R*_int_ = 0.054

#### Refinement

*R*[*F* ^2^ > 2σ(*F* ^2^)] = 0.048
*wR*(*F* ^2^) = 0.129
*S* = 1.13
3308 reflections
208 parameters
H-atom parameters constrained
Δρ_max_ = 0.86 e Å^−3^
Δρ_min_ = −0.73 e Å^−3^

### 

Data collection: *SMART* (Bruker, 1998 ▶); cell refinement: *SAINT-Plus* (Bruker, 1998 ▶); data reduction: *SAINT-Plus*; program(s) used to solve structure: *SHELXS97* (Sheldrick, 2008 ▶); program(s) used to refine structure: *SHELXL97* (Sheldrick, 2008 ▶); molecular graphics: *ORTEP-3* (Farrugia, 1997) ▶ and *CAMERON* (Watkin *et al.*, 1996) ▶; software used to prepare material for publication: *WinGX* (Farrugia, 1999 ▶).

## Supplementary Material

Crystal structure: contains datablocks global, I. DOI: 10.1107/S1600536811007343/pv2388sup1.cif

Structure factors: contains datablocks I. DOI: 10.1107/S1600536811007343/pv2388Isup2.hkl

Additional supplementary materials:  crystallographic information; 3D view; checkCIF report

## Figures and Tables

**Table 1:** Hydrogen-bond geometry (Å, °)

*D*—H⋯*A*	*D*—H	H⋯*A*	*D*⋯*A*	*D*—H⋯*A*
C1—H1*B*⋯N3^i^	0.97	2.57	3.423 (5)	147
C4—H4⋯N3^ii^	0.93	2.74	3.488 (5)	137

Symmetry codes: (i) 
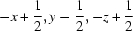
; (ii) 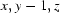.

**Fig. 1.**
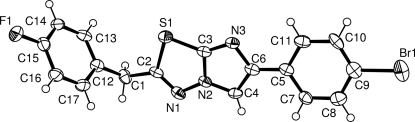
ORTEP-3 (Farrugia, 1997) view of the title compound, showing 50% probability ellipsoids and the atom numbering scheme.

**Fig. 2.**
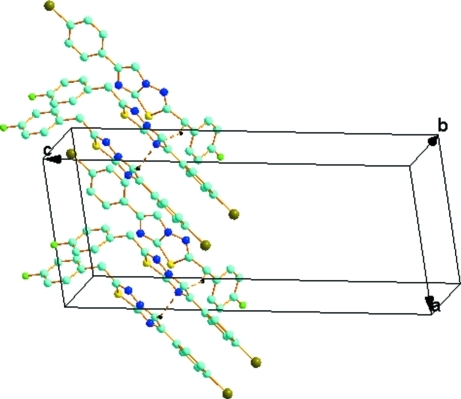
A part of the unit cell of the title compound showing intermolecular interactions with dotted lines. H-atoms not involved in hydrogen bonding have been excluded for clarity.

## supplementary crystallographic information

### Comment

Many imidazothiadiazole derivatives have been reported to possess diverse medicinal properties such as anthelmintic, antimicrobial, anti-inflammatory, antipyretic, analgesic properties and many other activities of therapeutic significance (Palagiano *et al.*, 1995). Moreover, the presence of fluoro substituent in a molecule enhances biological activity. Accumulation of fluorine on carbon leads to increased oxidative and thermal stability (Strunecka *et al.*, 2004; Park *et al.*, 2001). In this article we report the synthesis and crystal struuture of a novel imidazothiadiazole derivative, (I).

In the title compound (Fig. 1), the imidazothiadiazole and bromophenyl rings are individually planar with maximum deviations of 0.0215 (4) and 0.0044 (4) Å, for C2 and C9, respectively; the mean-planes of imidazothiadiazole and bromophenyl rings make a dihedral angle of 27.34 (3)° with respect to each other. Similar deviations from planarity of the corresponding rings have been reported earlier (Yang *et al.*, 2006). The dihedral angle between fluorobenzyl and imidazothiadiazole rings is 79.54 (3)° which is almost orthogonal. The thiadiazole moiety displays differences in the bond lengths S1-C2 [1.758 (4) Å] and S1-C3 [1.731 (4) Å] indicating that the resonance effect caused by the imidazole ring is stronger than that caused by the thiadiazole ring. The molecular structure is stabilized by strong (C1—H1B···N3) and ratherd weak (C4—H4···N3) intermolecular interactions resulting in chains of molecules lying along the *b*-axis (Table 1 and Fig. 2).

### Experimental

A mixture of equimolar quantities of 2-amino-(4-fluorobenyl)-1,3,4-thiadiazole (2.69, 0.013 mol) and phenacyl bromide (0.01 mol) was refluxed in dry ethanol for 18 hrs. The excess of solvent was distilled off and the solid hydrobromide salt that separated was collected by filtration, suspended in water and neutralized by aqueous sodium carbonate solution to get free base 6-(4-bromophenyl)-2-(4-fluorobenzyl)imidazo[2,1-*b*][1,3,4]thiadiazole. It was filtered, washed with water, dried and recrystallized from ethanol.

### Refinement

The H atoms were placed at calculated positions in the riding model approximation with C—H = 0.93 and 0.97 Å, for aryl and methylene type H-atoms, respectively, and *U*_iso_(H) = 1.2*U*_eq_(C).

### Crystal data

**Table:**

C_17_H_11_BrFN_3_S	*F*(000) = 776
*M**_r_* = 388.26	*D*_x_ = 1.690 Mg m^−^^3^
Monoclinic, *P*2_1_/*n*	Mo *K*α radiation, λ = 0.71073 Å
Hall symbol: -P 2yn	Cell parameters from 3308 reflections
*a* = 10.505 (4) Å	θ = 2.1–27.0°
*b* = 5.617 (2) Å	µ = 2.84 mm^−^^1^
*c* = 25.877 (11) Å	*T* = 296 K
β = 91.566 (7)°	Block, yellow
*V* = 1526.2 (11) Å^3^	0.18 × 0.16 × 0.16 mm
*Z* = 4	

### Data collection

**Table:**

Bruker SMART APEX CCD detector diffractometer	3308 independent reflections
Radiation source: fine-focus sealed tube	2419 reflections with *I* > 2σ(*I*)
graphite	*R*_int_ = 0.054
ω scans	θ_max_ = 27.0°, θ_min_ = 2.1°
Absorption correction: multi-scan (*SADABS*; Bruker, 1998)	*h* = −13→12
*T*_min_ = 0.629, *T*_max_ = 0.659	*k* = −6→7
8589 measured reflections	*l* = −31→33

### Refinement

**Table:**

Refinement on *F*^2^	Primary atom site location: structure-invariant direct methods
Least-squares matrix: full	Secondary atom site location: difference Fourier map
*R*[*F*^2^ > 2σ(*F*^2^)] = 0.048	Hydrogen site location: inferred from neighbouring sites
*wR*(*F*^2^) = 0.129	H-atom parameters constrained
*S* = 1.13	*w* = 1/[σ^2^(*F*_o_^2^) + (0.063*P*)^2^] where *P* = (*F*_o_^2^ + 2*F*_c_^2^)/3
3308 reflections	(Δ/σ)_max_ < 0.001
208 parameters	Δρ_max_ = 0.86 e Å^−^^3^
0 restraints	Δρ_min_ = −0.73 e Å^−^^3^

### Special details

**Table:**

Geometry. All s.u.'s (except the s.u. in the dihedral angle between two l.s. planes) are estimated using the full covariance matrix. The cell s.u.'s are taken into account individually in the estimation of s.u.'s in distances, angles and torsion angles; correlations between s.u.'s in cell parameters are only used when they are defined by crystal symmetry. An approximate (isotropic) treatment of cell s.u.'s is used for estimating s.u.'s involving l.s. planes.
Refinement. Refinement of *F*^2^ against ALL reflections. The weighted *R*-factor *wR* and goodness of fit *S* are based on *F*^2^, conventional *R*-factors *R* are based on *F*, with *F* set to zero for negative *F*^2^. The threshold expression of *F*^2^ > 2σ(*F*^2^) is used only for calculating *R*-factors(gt) *etc*. and is not relevant to the choice of reflections for refinement. *R*-factors based on *F*^2^ are statistically about twice as large as those based on *F*, and *R*- factors based on ALL data will be even larger.

### Fractional atomic coordinates and isotropic or equivalent isotropic displacement parameters (Å^2^)

**Table:**

	*x*	*y*	*z*	*U*_iso_*/*U*_eq_	
C1	0.1898 (4)	0.6339 (6)	0.36994 (13)	0.0319 (8)	
H1A	0.2138	0.4879	0.3877	0.038*	
H1B	0.1102	0.6044	0.3512	0.038*	
C2	0.2894 (3)	0.6920 (6)	0.33203 (13)	0.0278 (8)	
C3	0.4220 (3)	0.8748 (6)	0.26794 (13)	0.0264 (7)	
C4	0.5421 (3)	0.5636 (6)	0.25094 (13)	0.0277 (7)	
H4	0.5781	0.4124	0.2508	0.033*	
C5	0.6754 (3)	0.7708 (6)	0.18420 (13)	0.0253 (7)	
C6	0.5751 (3)	0.7565 (6)	0.22174 (13)	0.0258 (8)	
C7	0.7627 (3)	0.5856 (6)	0.17813 (13)	0.0291 (8)	
H7	0.7562	0.4509	0.1988	0.035*	
C8	0.8573 (3)	0.5950 (7)	0.14292 (14)	0.0335 (8)	
H8	0.9144	0.4694	0.1400	0.040*	
C9	0.8671 (3)	0.7945 (7)	0.11170 (14)	0.0342 (9)	
C10	0.7837 (3)	0.9808 (7)	0.11643 (14)	0.0358 (9)	
H10	0.7912	1.1151	0.0957	0.043*	
C11	0.6881 (3)	0.9679 (6)	0.15221 (14)	0.0323 (8)	
H11	0.6312	1.0939	0.1549	0.039*	
C12	0.1684 (3)	0.8245 (6)	0.40960 (14)	0.0281 (8)	
C13	0.0714 (3)	0.9915 (7)	0.40327 (14)	0.0336 (9)	
H13	0.0187	0.9835	0.3739	0.040*	
C14	0.0508 (3)	1.1678 (6)	0.43889 (14)	0.0321 (8)	
H14	−0.0145	1.2781	0.4341	0.038*	
C15	0.1308 (3)	1.1748 (6)	0.48200 (14)	0.0321 (8)	
C16	0.2275 (3)	1.0156 (7)	0.49006 (14)	0.0367 (9)	
H16	0.2803	1.0256	0.5194	0.044*	
C17	0.2456 (3)	0.8396 (6)	0.45377 (14)	0.0346 (9)	
H17	0.3107	0.7292	0.4590	0.042*	
N1	0.3702 (3)	0.5374 (5)	0.31627 (11)	0.0317 (7)	
N2	0.4447 (3)	0.6431 (5)	0.28024 (10)	0.0248 (6)	
N3	0.4997 (3)	0.9534 (5)	0.23239 (11)	0.0274 (6)	
S1	0.29995 (9)	0.97928 (16)	0.30528 (4)	0.0325 (2)	
F1	0.1127 (2)	1.3452 (4)	0.51794 (9)	0.0436 (6)	
Br1	0.99661 (4)	0.80778 (9)	0.062488 (15)	0.04750 (19)	

### Atomic displacement parameters (Å^2^)

**Table:**

	*U*^11^	*U*^22^	*U*^33^	*U*^12^	*U*^13^	*U*^23^
C1	0.039 (2)	0.033 (2)	0.0235 (18)	−0.0102 (16)	0.0011 (15)	0.0006 (15)
C2	0.0358 (19)	0.0223 (18)	0.0250 (18)	−0.0053 (15)	−0.0072 (15)	0.0042 (15)
C3	0.0315 (18)	0.0192 (17)	0.0279 (18)	−0.0006 (13)	−0.0079 (14)	0.0015 (14)
C4	0.0299 (18)	0.0196 (17)	0.0334 (19)	0.0015 (14)	−0.0052 (14)	0.0006 (15)
C5	0.0254 (17)	0.0286 (18)	0.0213 (17)	−0.0046 (14)	−0.0089 (13)	−0.0002 (14)
C6	0.0290 (18)	0.0197 (17)	0.0280 (19)	−0.0023 (13)	−0.0098 (14)	−0.0011 (14)
C7	0.0346 (19)	0.0265 (18)	0.0257 (18)	−0.0016 (15)	−0.0071 (15)	0.0010 (15)
C8	0.0309 (19)	0.036 (2)	0.033 (2)	0.0015 (16)	−0.0093 (15)	−0.0029 (17)
C9	0.0318 (19)	0.043 (2)	0.0275 (19)	−0.0122 (17)	−0.0065 (15)	−0.0046 (17)
C10	0.041 (2)	0.034 (2)	0.032 (2)	−0.0139 (17)	−0.0044 (16)	0.0087 (16)
C11	0.0339 (19)	0.0259 (19)	0.037 (2)	−0.0026 (15)	−0.0061 (16)	0.0064 (16)
C12	0.0257 (17)	0.0280 (19)	0.0303 (19)	−0.0032 (14)	−0.0011 (14)	0.0045 (15)
C13	0.0281 (18)	0.038 (2)	0.034 (2)	−0.0018 (16)	−0.0095 (15)	0.0107 (17)
C14	0.0289 (18)	0.035 (2)	0.032 (2)	0.0088 (16)	0.0037 (14)	0.0109 (16)
C15	0.0358 (19)	0.033 (2)	0.0276 (19)	0.0004 (16)	0.0081 (15)	0.0028 (16)
C16	0.037 (2)	0.042 (2)	0.030 (2)	0.0086 (17)	−0.0110 (16)	−0.0009 (17)
C17	0.0317 (19)	0.037 (2)	0.034 (2)	0.0110 (16)	−0.0053 (15)	0.0007 (16)
N1	0.0389 (17)	0.0267 (16)	0.0293 (17)	−0.0049 (13)	−0.0045 (13)	0.0036 (13)
N2	0.0315 (15)	0.0229 (15)	0.0198 (14)	−0.0028 (11)	−0.0027 (11)	0.0037 (11)
N3	0.0318 (15)	0.0231 (15)	0.0271 (15)	0.0007 (12)	−0.0040 (12)	0.0045 (12)
S1	0.0370 (5)	0.0238 (5)	0.0368 (5)	0.0019 (4)	0.0014 (4)	0.0039 (4)
F1	0.0542 (14)	0.0404 (13)	0.0364 (13)	0.0138 (11)	0.0056 (10)	−0.0061 (10)
Br1	0.0394 (3)	0.0716 (4)	0.0315 (2)	−0.0207 (2)	0.00146 (17)	−0.0057 (2)

### Geometric parameters (Å, °)

**Table:**

C1—C2	1.490 (5)	C8—H8	0.9300
C1—C12	1.504 (5)	C9—C10	1.373 (5)
C1—H1A	0.9700	C9—Br1	1.890 (4)
C1—H1B	0.9700	C10—C11	1.386 (5)
C2—N1	1.288 (4)	C10—H10	0.9300
C2—S1	1.761 (3)	C11—H11	0.9300
C3—N3	1.322 (4)	C12—C17	1.386 (5)
C3—N2	1.360 (4)	C12—C13	1.391 (5)
C3—S1	1.729 (4)	C13—C14	1.374 (5)
C4—N2	1.365 (4)	C13—H13	0.9300
C4—C6	1.371 (5)	C14—C15	1.379 (5)
C4—H4	0.9300	C14—H14	0.9300
C5—C11	1.391 (5)	C15—F1	1.351 (4)
C5—C7	1.399 (5)	C15—C16	1.366 (5)
C5—C6	1.455 (5)	C16—C17	1.380 (5)
C6—N3	1.392 (4)	C16—H16	0.9300
C7—C8	1.368 (5)	C17—H17	0.9300
C7—H7	0.9300	N1—N2	1.369 (4)
C8—C9	1.387 (5)		
			
C2—C1—C12	114.5 (3)	C9—C10—C11	119.7 (3)
C2—C1—H1A	108.6	C9—C10—H10	120.1
C12—C1—H1A	108.6	C11—C10—H10	120.1
C2—C1—H1B	108.6	C10—C11—C5	121.5 (3)
C12—C1—H1B	108.6	C10—C11—H11	119.3
H1A—C1—H1B	107.6	C5—C11—H11	119.3
N1—C2—C1	122.8 (3)	C17—C12—C13	117.8 (3)
N1—C2—S1	116.4 (3)	C17—C12—C1	120.7 (3)
C1—C2—S1	120.8 (3)	C13—C12—C1	121.5 (3)
N3—C3—N2	112.0 (3)	C14—C13—C12	122.4 (3)
N3—C3—S1	139.2 (3)	C14—C13—H13	118.8
N2—C3—S1	108.8 (2)	C12—C13—H13	118.8
N2—C4—C6	104.6 (3)	C13—C14—C15	117.4 (3)
N2—C4—H4	127.7	C13—C14—H14	121.3
C6—C4—H4	127.7	C15—C14—H14	121.3
C11—C5—C7	116.8 (3)	F1—C15—C16	118.5 (3)
C11—C5—C6	121.6 (3)	F1—C15—C14	118.9 (3)
C7—C5—C6	121.5 (3)	C16—C15—C14	122.7 (3)
C4—C6—N3	111.4 (3)	C15—C16—C17	118.7 (3)
C4—C6—C5	127.6 (3)	C15—C16—H16	120.6
N3—C6—C5	121.0 (3)	C17—C16—H16	120.6
C8—C7—C5	122.5 (3)	C16—C17—C12	121.1 (3)
C8—C7—H7	118.8	C16—C17—H17	119.4
C5—C7—H7	118.8	C12—C17—H17	119.4
C7—C8—C9	119.1 (4)	C2—N1—N2	108.5 (3)
C7—C8—H8	120.5	C3—N2—C4	108.2 (3)
C9—C8—H8	120.5	C3—N2—N1	118.3 (3)
C10—C9—C8	120.4 (4)	C4—N2—N1	133.5 (3)
C10—C9—Br1	120.2 (3)	C3—N3—C6	103.8 (3)
C8—C9—Br1	119.4 (3)	C3—S1—C2	87.94 (17)
			
C12—C1—C2—N1	139.7 (3)	C13—C14—C15—C16	0.1 (6)
C12—C1—C2—S1	−41.6 (4)	F1—C15—C16—C17	179.3 (3)
N2—C4—C6—N3	−0.1 (4)	C14—C15—C16—C17	−0.4 (6)
N2—C4—C6—C5	179.0 (3)	C15—C16—C17—C12	0.7 (6)
C11—C5—C6—C4	171.4 (3)	C13—C12—C17—C16	−0.5 (6)
C7—C5—C6—C4	−7.8 (5)	C1—C12—C17—C16	179.2 (3)
C11—C5—C6—N3	−9.6 (5)	C1—C2—N1—N2	177.4 (3)
C7—C5—C6—N3	171.3 (3)	S1—C2—N1—N2	−1.3 (4)
C11—C5—C7—C8	0.5 (5)	N3—C3—N2—C4	−0.5 (4)
C6—C5—C7—C8	179.7 (3)	S1—C3—N2—C4	−179.3 (2)
C5—C7—C8—C9	−0.5 (5)	N3—C3—N2—N1	−179.4 (3)
C7—C8—C9—C10	0.6 (5)	S1—C3—N2—N1	1.8 (3)
C7—C8—C9—Br1	−179.4 (2)	C6—C4—N2—C3	0.3 (3)
C8—C9—C10—C11	−0.8 (5)	C6—C4—N2—N1	179.0 (3)
Br1—C9—C10—C11	179.2 (3)	C2—N1—N2—C3	−0.4 (4)
C9—C10—C11—C5	0.9 (5)	C2—N1—N2—C4	−178.9 (3)
C7—C5—C11—C10	−0.7 (5)	N2—C3—N3—C6	0.4 (4)
C6—C5—C11—C10	−179.9 (3)	S1—C3—N3—C6	178.7 (3)
C2—C1—C12—C17	−83.6 (4)	C4—C6—N3—C3	−0.2 (4)
C2—C1—C12—C13	96.2 (4)	C5—C6—N3—C3	−179.4 (3)
C17—C12—C13—C14	0.2 (5)	N3—C3—S1—C2	179.7 (4)
C1—C12—C13—C14	−179.6 (3)	N2—C3—S1—C2	−1.9 (2)
C12—C13—C14—C15	0.1 (5)	N1—C2—S1—C3	2.0 (3)
C13—C14—C15—F1	−179.7 (3)	C1—C2—S1—C3	−176.8 (3)

### Hydrogen-bond geometry (Å, °)

**Table:**

*D*—H···*A*	*D*—H	H···*A*	*D*···*A*	*D*—H···*A*
C1—H1B···N3^i^	0.97	2.57	3.423 (5)	147
C4—H4···N3^ii^	0.93	2.74	3.488 (5)	137

Symmetry codes: (i) −*x*+1/2, *y*−1/2, −*z*+1/2; (ii) *x*, *y*−1, *z*.
